# Replacement of corn with rice grains did not alter growth performance and rumen fermentation in growing Hanwoo steers

**DOI:** 10.5713/ajas.19.0691

**Published:** 2019-12-13

**Authors:** Sungjae Yang, Byeongwoo Kim, Hanbeen Kim, Joonbeom Moon, Daekyum Yoo, Youl-Chang Baek, Seyoung Lee, Jakyeom Seo

**Affiliations:** 1 Department of Animal Science, Life and Industry Convergence Research Institute, Pusan National University, Miryang 50463, Korea; 2 National Institute of Animal Science, Rural Development Administration, Wanju 55365, Korea; 3 Division of Animal Husbandry, Yonam College, Cheonan 31005, Korea

**Keywords:** Rice Grains, Total Mixed Rations, *In vitro* Fermentation, Growth Performances

## Abstract

**Objective:**

This study was realized to evaluate the nutritional value of rice grains as a replacement for corn grains in the diet of growing Hanwoo steers.

**Methods:**

Two experimental diets were prepared: i) Corn total mixed ration (TMR) consisting of 20% corn grains and ii) Rice TMR consisting of 20% rice grains, in a dry matter (DM) basis. These treatments were used for *in vitro* rumen fermentation and *in vivo* growth trials. In the rumen fermentation experiment, the *in vitro* DM digestibility (IVDMD), *in vitro* crude protein digestibility (IVCPD), *in vitro* neutral detergent fiber digestibility, pH, ammonia nitrogen, and volatile fatty acids (VFA) were estimated at 48 h, and the gas production was measured at 3, 6, 12, 24, and 48 h. Twenty four growing Hanwoo steers (9 months old; body weight [BW]: 259±13 kg) were randomly divided into two treatment groups and the BW, dry matter intake (DMI), average daily gain (ADG), and feed conversion ratio (FCR) were measured.

**Results:**

The *in vitro* experiment showed that the IVDMD, IVCPD, and VFA production of the Rice TMR were higher than those of the Corn TMR (p<0.05). The growth trial showed no differences (p>0.05) in the final BW, ADG, DMI, and FCR between the two TMRs.

**Conclusion:**

The use of rice grains instead of corn grains did not exhibit any negative effects on the rumen fermentation or growth performance, thereby rice grains with a DM of less than 20% could be used as a starch source in the diet of growing steers.

## INTRODUCTION

Corn grains are used as a primary energy source for ruminants. In South Korea, all corn grains used as feed ingredients have been dependent upon imports, illustrating one of the major reasons for consistently high feed costs.

Rice grains, which account for about 20% of the world’s grain production, are the third most commonly produced grain, followed by corn and wheat, and are mainly cultivated in Asia [1]. The nutritive values of rice grains (crude protein [CP], 7.06%; ether extract [EE], 3.02%; nitrogen free extract [NFE], 87.17%; and total digestible nutrients [TDN], 88.37%) were similar to those of corn grains (CP, 8.46%; EE, 4.00%; NFE, 83.86%; and TDN, 89.44%) [2]. On the basis of the similar nutritional values of the two grains, rice grains have been considered as a replacement for corn grains, but rice has rarely been used as a feed ingredient due to its recognition as a food resource in Korea.

Recently, as the westernization of lifestyle has caused a decrease in the consumption of rice, many studies using rice grains as a feed source have been conducted in Asian countries. In Japan, rice grains have been used as a feed ingredient for livestock since the 1990s, with Miyaji et al [3] reporting the possibility of using rice grains to replace corn grains in total mixed rations (TMR) through an *in vivo* study on dairy cattle. In South Korea, rice grains have been evaluated as a feed ingredient since the 2000s [4,5]. Recently, the nutritional values of rice grains processed in different ways have been assessed [6]. Yang et al [7] also reported that there were no negative effects on the characteristics of rumen fermentation upon replacing corn grains with rice grains at about 70% in the TMR in an *in vitro* system.

Previous studies concerning the use of rice grains as a substitute for corn grains were mainly carried out using dairy cattle. To our knowledge, there are few studies that have been conducted on beef cattle. Therefore, the purpose of this study was to evaluate the utilization of rice grains as a main feed ingredient for growing Hanwoo steers.

## MATERIALS AND METHODS

The protocols for this study concerning animal use were reviewed and approved by the Animal Research Ethics Committee of Pusan National University (Pusan, Korea, PNU-2019-2239).

### Preparation of experimental diets and chemical analysis

To prepare two experimental TMRs, a commercial concentrate mix (Nonghyup Feed, Co. Ltd., Miryang, Korea), alfalfa, corn, and rice grains were used as the main ingredients. The TMR containing 20% flaked corn was used as the control diet (Corn TMR) and the TMR containing 20% rice grains was used as the treatment diet (Rice TMR) for the *in vitro* and *in vivo* experiments. Both diets were formulated to meet the adequate metabolizable energy and metabolizable protein for growing beef cattle, with an average daily gain (ADG) of 1,000 g/d [8]. The formulation and chemical composition of the experimental diets are shown in Table 1. Before conducting the chemical analysis, all feed ingredients and experimental diets were dried at 60°C for 96 h and ground using a cyclone mill (Foss Tecator Cyclotec 1093, Foss, Hillerød, Denmark) fitted with a 1 mm screen. The dry matter (DM, #934.01), CP (#976.05), EE (#920.39), acid detergent fiber (#973.18), and ash (#942.05) were analyzed using AOAC international methods [9]. The crude protein was calculated by multiplying the nitrogen contents by 6.25. The total nitrogen was measured using a nitrogen combustion analyzer (Leco FP-528 Leco, St. Joseph, MI, USA) according to the Kjeldahl method. The neutral detergent fiber (aNDF) and lignin were analyzed by utilizing the methods described by Van Soest et al [10] to determine fiber contents. Heat-stable amylase (α-amylase) was used for aNDF estimation and expressed inclusive of residual ash. The TDN and net energy for maintenance of the experimental diets were estimated based on the equations in the NRC [8,11].

### *In vitro* fermentation

*In vitro* fermentation was carried out using rumen fluid collected from two cannulated Holsteins (body weight [BW]: 450±30 kg) before morning feeding at the Center for Agriculture Research, Pusan National University, Korea. Animals were fed a diet consisting of 600 g/kg of timothy hay and 400 g/kg of a commercial concentrate mix. The respective rumen fluids collected before the morning feeding time mixed together, transferred into a thermos bottle, and immediately transported to the laboratory. The rumen contents were filtered through 8 layers of cheesecloth and mixed with 2 volumes of *in vitro* rumen buffer solution, which was done in accordance with a modified method described by Goering and Van Soest [12] under strictly anaerobic conditions. Approximately 1 g of the ground experimental substrates was put into pre-weighed filter bags (F57, Ankom Technology, Macedon, NY, USA). All bags were heat-sealed and transferred into empty 500 mL Duran bottles. Five bottles were used for each dietary treatment and 2 bags were stored per bottle. Then, 250 mL of rumen fluid and buffer mixture was transferred to each bottle, accompanied by continuous flushing with O_2_-free CO_2_ gas. The bottles were sealed with caps and incubated on a rotary shaker (JSSI-300T, JS Research Inc., Gongju, Korea) for 48 h at 125 rpm and 39°C. After 48 h of incubation, the gas production, DM digestibility (IVDMD), CP digestibility (IVCPD), pH, ammonia nitrogen (NH_3_-N), and concentration of volatile fatty acids (VFA) were measured. The gas production was measured at 3, 6, 12, 24, and 48 h using a pressure transducer (Sun Bee Instrument Inc., Seoul, Korea), as described by Theodorou et al [13]. Gas production profiles obtained during incubation were fitted to a simple exponential model [14], the equation for which is as follows:

$$\begin{array}{ll} V_{T}=0 & \left(0\leq \text{T}\leq \text{L}\right)\\ V_{T}=V_{\text{max}}\times (1-e^{\left[-kg\times (T-L)\right]}) & \left(\text{T}\geq \text{L}\right) \end{array}$$

where *T* is the time (h), *L* is the lag time (h), *e* is the exponential function, *k_g_* is the fractional rate of gas production (h^−1^), *V*_max_ is the theoretical maximum gas production (mL) after the asymptote is reached, and VT is the gas produced at time T (mL). After incubation, the bottle caps were removed; then, the bottles were immediately placed on ice to stop the fermentation. The filter bags were removed from the bottles and rinsed with flowing water until the water ran clear. The washed bags were dried at 55°C for 48 h, and then weighed to measure the IVDMD. The IVCPD was estimated using Kjeldahl nitrogen analysis. The aNDF content was assessed for the weighed bags using a modified version of the micro-NDF method proposed by Pell and Schofield [15] to evaluate aNDF digestibility (IVNDFD). The pH of the culture fluid was measured using a pH meter (FP20, Mettler Toledo, Columbus, OH, USA). The culture fluid was centrifuged at 15,000×g for 10 min at 4°C, and then stored at −20°C until the analysis of the VFA and NH_3_-N concentrations.

For the VFA analysis, 200 μL of the supernatant was diluted with 800 μL of ethanol after centrifugation at 20,000 rcf for 15 min. The VFA concentration was measured using a gas chromatograph (Agilent 7890A, Agilent Technology, Santa Clara, CA, USA) equipped with a flame ionization detector and capillary column (Nukol Fused silica capillary column, 30 m×250 μm×0.25 μm, Supelco Inc., Bellefonte, PA, USA). The temperatures of the oven, injector, and detector were set at 90°C, 90 to 200°C, and 230°C, respectively. Nitrogen was used as the carrier gas, at a flow rate of 30 mL/min. The ammonia concentration was analyzed following the methods describe by Chaney and Marbach [16] with several modifications. After 2 μL of the supernatant was mixed with 100 μL of a phenol color reagent (50 g of phenol, 0.25 g of sodium nitroferricyanide, and 1 L of distilled water) and alkali hypochlorite (25 g of sodium hydroxide, 16.8 mL of sodium hydroxide, and 1 L of distilled water), the mixture was incubated in a 37°C water bath for 15 min. The ammonia concentration was determined by measuring the absorbance at 630 nm using a microplate reader (iMARK, Biorad, Hercules, CA, USA).

### *In vivo* experimental design

A total of 24 Hanwoo steers (9 months old; BW 259±13 kg) were randomly allocated to the following two dietary treatment groups: i) Corn TMR and ii) Rice TMR. Each treatment had 3 replicated pens with 4 steers per pen, which was equipped with a feeder and automated drinker. Therefore, each pen was considered as an experimental unit. All diets were prepared weekly and provided to the steers once per day at the feeding time (0800) *ad libitum* (targeting 10% refusal). The experiment lasted for 9 weeks. Feeds offered and refused in each pen were measured daily and calculated as the DM for daily DM intake (DMI). On the first and last days of the experiment, all steers were weighed to measure the ADG and feed conversion ratio (FCR).

### Statistical analysis

The data from the *in vitro* and *in vivo* experiments were analyzed using the general linear model procedure of the SAS package 9.4 (SAS Institute Inc., Carey, NC, USA). The general linear model for the statistical analysis is as follows:

$$y_{ij}=\mu +\tau_{i}+e_{ij}$$

where *y_ij_* is the *j*th observation in the *i*th treatment, *μ* is the overall mean, *τ_i_* is the fixed effect of the *i*th treatment, and *e_ij_* is the unexplained random effect on the *j*th observation for the *i*th treatment. Differences between treatments were analyzed using the Tukey test, with statistical significance declared at p<0.05 and a trend discussed at 0.05≤p<0.1.

## RESULTS

The chemical compositions of the Corn and Rice TMRs were similar (Table 1) because the nutrient compositions of corn and rice did not differ (data not shown).

*In vitro* fermentation experiments, the IVDMD and IVCPD were higher (p<0.05) in the Rice TMR than in the Corn TMR (Table 2), although there was no difference in the IVNDFD. Compared to the Corn TMR, the use of Rice TMR increased (p<0.05) the total VFA concentration, without causing any changes in the proportions of VFAs. Therefore, the acetate to propionate ratio did not differ (p>0.10) between the two experimental diets. The pH, NH_3_-N, and gas production measured at each time point were not affected (p>0.10) by the substitution of rice in the TMR (Table 2). In the gas profiles, *V*_max_ showed a higher tendency in the Rice TMR than in the Corn TMR (p = 0.07); however, *k_g_* tended to be higher in the Corn TMR (p = 0.06, Table 2).

The data concerning the growth performance of growing Hanwoo steers fed each experimental diet were shown in Table 3. During the experimental period, the change in the BW was 57.4±9.5 kg and 55±15.7 kg for the Corn and Rice TMRs, respectively. Differences in the initial BW, final BW, ADG, DMI, and FCR were not observed.

## DISCUSSION

Because heat treatment can induce the gelatinization of starch structures and this can lead to higher digestibility in the rumen [17], we expected that the Corn TMR containing flaked corn would have a higher digestibility than the Rice TMR, which contained no-heat treated rice. However, the results from the *in vitro* fermentation revealed that the substitution of rice grains for corn grains in TMR improved the rumen digestibility and VFA concentration. This result is similar to those of previous studies that have highlighted the higher digestibility of rice grains in the ruminal environment [3–5]. Additionally, in an *in vitro* experiment, Yang et al [7] reported that the DM digestibility of a TMR containing 70% rice grains was higher than that of a TMR containing corn grains.

Protein is one of the essential nutrients for promoting ruminal fermentation and the formation of body tissues on beef cattle [18,19]. According to the present study, the IVCPD was higher for the Rice TMR, although the two dietary treatments showed similar CP contents. Yang et al [7] reported that rice grains, used to replace corn grains in an *in vitro* experiment, did not induce a significant difference in CP digestibility. Additionally, *in vivo* studies have reported that the replacement of corn grains with rice grains does not cause a significant difference in the whole tract CP digestibility [3,5,20–22].

Ammonia nitrogen, generated by the microbial fermentation of protein sources in feed, is dependent upon an intimate relationship between protein availability within the rumen microorganism and the microbial conversion efficiency [23]. In our *in vitro* experiment, the rice treatment may have improved the protein availability in the rumen, considering that the IVCPD increased without a significant change in the NH_3_-N concentration when using 20% DM of rice grains in the TMR.

In the *in vitro* experiment, the rice treatment improved the total VFA concentrations, which are considered as final products of rumen fermentation, without causing significant differences in the individual VFA compositions. Previous studies have consistently reported that using rice grains as a replacement for corn grains does not cause a significant difference in the total VFA concentration, although there were inconsistent changes on the compositions of individual VFAs [3,5,21]. Miyaji et al [3,21] reported that the proportion of acetate decreased, whilst that of propionate increased, upon replacing corn with rice grains at below 40% in an *in vivo* trials. Oh et al [5] reported that there was an increase in the proportion of acetate and decrease in the proportion of propionate when rice grains were used at 50% of the feed intake in an *in vivo* experiment.

In the feeding trials conducted in this study, the use of present proportion of rice grains did not result in negative effects on growth performances. Whilst many previous studies have reported that increasing the proportion of rapidly available starch sources in the feed could decrease the DMI [24,25], we observed no difference in the DMI, even though the proportion of starch was higher in the rice grains than in the corn grains used in the present study (rice starch, 78.1% DM; corn starch, 71.5% DM, data not shown). This is consistent with the previous studies that have reported that the DMI did not exhibit significant differences in dairy cattle when using rice grains instead of corn grains in the feed containing approximately 31% DM [3] and 36% DM [22].

Studies substituting corn grains with rice grains have been carried out not only in ruminants but also in monogastric animals [26–28]. Feeding 50% and 96.6% (as-fed basis) of rice grains instead of corn grains to pigs caused a higher DM digestibility, without changing the CP digestibility [26,27]. Nanto et al [28] reported that the BW, feed intake, and FCR were similar when broilers were fed dehulled rice grains as the main cereal source at over 42% (as-fed basis) in the feed ration (p> 0.10). On the contrary, Miyaji et al [21] stated that the substitution of corn grains with rice grains at 40% DM in the feed rations caused negative effects on the DMI, milk yield, and milk protein yield, although it improved the total digestibility and starch digestibility in dairy cattle. It is speculated that the differences in results between ruminants and monogastric animals are due to the distinctive types of digestive organs in these animals, especially the rumen, in which the microbial digestion of feed predominantly turns fermentable substrates into organic acids [29]. The effects on productivity could also differ according to the proportion of rice grains, processing treatment, and ruminant type [30].

Overall, our results highlight that using rice grains in TMRs at a 20% DM level has no negative effects on growth performances and rumen fermentation in growing Hanwoo steers. Further studies are required to investigate the proper proportion of rice grains in feed for beef cattle during the finishing period.

## CONCLUSION

In the feeding trial, none of the growth performances (BW, ADG, DMI, and FCR) showed significant differences between the two treatments. In terms of the rumen fermentation characteristics, the IVDMD, IVCPD, and VFA were higher in the Rice TMR, but there were no significant changes on pH, NH_3_-N, IVNDFD, and gas production between the two treatments. This means that using rice grains could be considered a substitution feedstuff for corn grains in TMRs at a 20% DM level.

## Figures and Tables

**Table 1:** Diet formulation and chemical composition (% dry matter or as stated) of the experimental diets

Items	Treatments1)	

	Corn TMR	Rice TMR
Ingredients (% DM)		
Corn flake	20.0	0.00
Rice grain	0.00	20.0
Commercial concentrate mix	44.5	44.5
Alfalfa	35.2	35.2
Vitamin and mineral mix2)	0.3	0.3
Chemical composition, dry matter or as stated		
DM (% as fed)	65.00	65.00
CP	16.40	16.36
aNDF	28.53	27.87
ADF	18.50	17.92
Lignin	5.33	5.15
EE	3.26	3.07
Ash	6.80	6.93
TDN	70.78	71.6
NEm (Mcal/kg of DM)	1.62	1.64

DM, dry matter; CP, crude protein; aNDF, neutral detergent fiber analyzed with heat stable α-amylase; ADF, acid detergent fiber; EE, ether extract; TDN, total digestible nutrients; NEm, net energy for maintenance.

1) Corn TMR, control diet containing 20% flaked corn; Rice TMR, substitute diet containing 20% rice grain

2) 33,330,000 IU/kg of vitamin A, 40,000,000 IU/kg of vitamin D, 20.86 IU/kg of vitamin E, 20 mg/kg of Cu, 90 mg/kg of Mn, 100 mg/kg of Zn, 250 mg/kg of Fe, 0.4 mg/kg of I, and 0.4 mg/kg of Se.

**Table 2:** *In vitro* fermentation characteristics of the experimental diets at 48 h of incubation

Items	Treatments1)		SEM	p-value

	Corn TMR	Rice TMR		
Rumen parameters				
IVDMD (%)	69.7^b^	71.0^a^	0.502	<0.05
IVCPD (% CP)	83.8^b^	85.1^a^	0.531	<0.05
IVNDFD (% aNDF)	41.1	33.3	6.417	0.27
pH	6.35	6.34	0.017	0.66
NH_3_-N (mg/dL)	36.1	35.5	0.759	0.42
TVFA (mM)	74.2^b^	79.8^a^	2.282	<0.05
Acetate (mmol/mol)	468.6	465.4	2.759	0.28
Propionate (mmol/mol)	301.6	295.8	3.860	0.17
Butyrate (mmol/mol)	139.0	145.6	3.435	0.09
A:P ratio	1.56	1.57	0.026	0.51
Gas (mL/g DM)				
3 h	25.3	24.4	2.090	0.66
6 h	40.4	37.9	1.985	0.26
12 h	71.0	68.0	2.243	0.21
24 h	134.7	133.0	3.212	0.62
48 h	204.1	205.3	3.527	0.74
Fitted parameters of gas2)				
*V*_max_	299.8	326.6	12.99	0.07
*k*_g_	0.024	0.021	0.001	0.06

SEM, standard error of the mean; IVDMD, *in vitro* dry matter digestibility; IVCPD, *in vitro* crude protein digestibility; IVNDFD, *in vitro* neutral detergent fiber digestibility; NH_3_-N, ammonia; TVFA, total volatile fatty acids; A:P ratio, acetate to propionate ratio; DM, dry matter.

1) Corn TMR, control diet containing 20% flaked corn; Rice TMR, substitute diet containing 20% rice grain.

2) *V*_max_, theoretical maximum gas production (mL/g DM); and *k*_g_, fractional rate of gas production (h^−1^).

a,b Values in the same row with different letters indicate significant differences (p<0.05).

**Table 3:** *In vivo* growth performances for the experimental diets

Items	Treatments1)		SEM	p-value

	Corn TMR	Rice TMR		
Initial BW (kg)	259.0	258.8	5.509	0.98
Final BW (kg)	316.4	313.8	8.704	0.77
ADG (g/d)	902.5	857.8	68.54	0.52
DMI (kg/d)	7.36	7.35	0.302	0.96
FCR1)	9.25	9.91	0.822	0.43

SEM, standard error of the mean; BW, body weight; ADG, average daily gain; DMI, dry matter intake; FCR, feed conversion ratio (DMI/ADG).

1) Corn TMR, control diet containing 20% flaked corn; Rice TMR, substitute diet containing 20% rice grain.
